# Supplementary material for: Novel RNA and DNA strand exchange activity of the PALB2 DNA binding domain and its critical role for DNA repair in cells
Source: eLife. 2019 Apr 29;8:e44063. doi: 10.7554/eLife.44063 (PMC6533086; doi:10.7554/eLife.44063)
Supplement: Figure 1—source data 1. [file elife-44063-fig1-data1.docx]

|  | **T1** | **T3** | **573** |
| --- | --- | --- | --- |
| **ss20** | | | |
| **n** | 1.1±0.1 | 1.8±0.3 | 0.9±0.1 |
| **Kd (nM)** | 80±8.5 | 191±20 | 50±7.3 |
| **ds20** | | | |
| **n** | 1.0±0.1 | 5.4±2 | 0.9±0.1 |
| **Kd (nM)** | 28.6±13 | 108±64 | 43±5.6 |
| **ss49** | | | |
| **n** | 1.3±0.1 | 1.3±0.2 | 0.7±0.1 |
| **Kd (nM)** | 4.0±1.3 | 484±80 | 4.8±0.4 |
| **ds49** | | | |
| **n** | 3.2±0.4 | 1.2±0.1 | 1.1± 0.1 |
| **Kd (nM)** | 23±9 | 222±10 | 44±6 |
