# Supplementary material for: Novel RNA and DNA strand exchange activity of the PALB2 DNA binding domain and its critical role for DNA repair in cells
Source: eLife. 2019 Apr 29;8:e44063. doi: 10.7554/eLife.44063 (PMC6533086; doi:10.7554/eLife.44063)
Supplement: Figure 2—source data 1. [file elife-44063-fig2-data1.docx]

|  | **T1** | **T1 RK** | **T1 RRKK** | **573** | **573 RRKK** |
| --- | --- | --- | --- | --- | --- |
| **n** | 1.3 | 0.9 | 0.8 | 0.7 | 1.6 |
| **Kd (nM)** | 4.0±1.3 | 285±71 | 316±59 | 4.8±0.4 | 187±55 |
