# Supplementary material for: Novel RNA and DNA strand exchange activity of the PALB2 DNA binding domain and its critical role for DNA repair in cells
Source: eLife. 2019 Apr 29;8:e44063. doi: 10.7554/eLife.44063 (PMC6533086; doi:10.7554/eLife.44063)
Supplement: Supplementary file 1. — (A) DNA binding parameters of N-DBD mutants, and DNA/RNA sequences for (B) primers for DNA binding site mutagenesis, (C) substrates for DNA binding assay, (D) Substrates for strand exchange activity and FRET assays, (E) siRNA resistance primers. [file elife-44063-supp1.docx]

**Table A.** Equilibrium dissociation constants and n values for PALB2 mutants binding experiment in Figure 2-figure supplement 2.

|  | **T1** | **K45A** | **K50A** | **KK45AA** | **R170A** | **K174A** | **RR170174AA** | **RK170175AA** | **RKR170AAA** |
| --- | --- | --- | --- | --- | --- | --- | --- | --- | --- |
| **ss49** |  |  |  |  |  |  |  |  |  |
| n | 1.2 | 0.7 | 0.8 | 1.1 | 0.9 | 0.8 | 1 | 0.898 | 0.67 |
| Kd | 4.0±1.3 | 65.3±1.1 | 7.9±1.0 | 28.6±1.2 | 24.6±1.1 | 19.7±1.2 | 10.4±1.0 | 28.2±1.1 | 29.5±1.3 |
| **ss25** |  |  |  |  |  |  |  |  |  |
| n | 1 | 0.8 | 0.8 | 0.8 | 0.7 | 0.8 | 0.6 | 0.7 | 0.6 |
| Kd | 41.7±1.0 | 476±1.1 | 94.9±1.0 | 222±1.3 | 1490±1.0 | 54.7±1.2 | 884±1.2 | 889±1.4 | 2403±1.5 |

**Table B.** Primers for DNA binding site mutagenesis.

| **Mutation** | **Sequence** |
| --- | --- |
| RK146AA F | ACT GCC GAG CGC TCG TGC AAA ACA ACA AAA GC |
| RK146AA R | GCT TTT GTT GTT TTG CAC GAG CGC TCG GCA GT |
| RK147AA F | ACT GCC GAG CCG TGC TAA AGC ACA ACA AAA GC |
| RK147AA R | GCT TTT GTT GTG CTT TAG CAC GGC TCG GCA GT |
| KKK90AAA F | CGA AAA AAT TGC ACA TAG CAT TGC AGC AAC GGT GGA AG |
| KKK90AAA R | CTT CCA CCG TTG CTG CAA TGC TAT GTG CAA TTT TTT CG |
| K90A F | CGA AAA AAT TGC ACA TAG CAT TAA AAA AAC |
| K90A R | GTT TTT TTA ATG CTA TGT GCA ATT TTT TCG |
| K94A F | AAT TAA ACA TAG CAT TGC AAA AAC GGT GGA AG |
| K94A R | CTT CCA CCG TTT TTG CAA TGC TAT GTT TAA TT |
| K95A F | CAT AGC ATT AAA GCA ACG GTG GAA GAA C |
| K95A R | GTT CTT CCA CCG TTG CTT TAA TGC TAT G |
| RKR190AAA F | CCG ATT CGT TAG CTC TGA GCG GCG CAG CGC TGA AAG AAC |
| RKR190AAA R | GTT CTT TCA GCG CTG CGC CGC TCA GAG CTA ACG AAT CGG |
| R190A F | CCG ATT CGT TAG CTC TGA GCG G |
| R190A R | CCG CTC AGA GCT AAC GAA TCG G |
| K194A F | CTG AGC GGC GCA CGG CTG AAA GAA C |
| K194A R | GTT CTT TCA GCC GTG CGC CGC TCA G |
| R195A F | CTG AGC GGC AAA GCG CTG AAA GAA CAG |
| R195A R | CTG TTC TTT CAG CGC TTT GCC GCT CAG |
| KR174,175A F | CTG AGC GGC GCA GCG CTG AAA GAA CAG |
| KR174,175A R | CTG TTC TTT CAG CGC TGC GCC GCT CAG |
| KK49,50AA F | A ATT AAA CAT AGC ATT GCA GCA ACG GTG GAA G |
| KK49,50AA R | CTT CCA CCG TTG CTG CAA TGC TAT GTT TAA TT |
| RRKK146AAAA F | AAA CTG CCG AGC GCT GCT GCA GCA CAA CAA AAG CGC |
| RRKK146AAAA R | GCG CTT TTG TTG TGC TGC AGC AGC GCT CGG CAG TTT |
| K148A F | GAG CCG TCG TGC CAA ACA ACA AAA G |
| K148A R | CTT TTG TTG TTT GGC ACG ACG GCT C |
| R146A F | ACT GCC GAG CGC TCG TAA AAA AC |
| R146A R | GTT TTT TAC GAG CGC TCG GCA GT |
| K148A – RK147 | GAG CCG TGC TGC CGC ACA ACA AAA G |
| K148A – RK147 | CTT TTG TTG TGC GGC AGC ACG GCT C |
| R146A –RK147 | ACT GCC GAG CGC CGC TAA AGC AC |
| R146A –RK147 | GTG CTT TAG CGG CGC TCG GCA GT |
| RK146,148AA –RK147 | ACT GCC GAG CGC CGC TGC GGC ACA ACA AAA G |
| RK146,148AA –RK147 | CTT TTG TTG TGC CGC AGC GGC GCT CGG CAG T |
| 146AAAA (JYM3909F) | GCGGCGCAGCAGAAGAGGACATTTATTTC |
| 146AAAA (JYM3910R) | TGCTGCGCTTGGCAGCTTCTGCTT |

**Table C.** Substrates for DNA binding assay.

| **Sample** | **Sequence** |
| --- | --- |
| **15-FAM** | /56-FAM/-CCGCTACCAGTGATC |
| **15-comp** | GATCACTGGTAGCGG |
| **20-FAM** | /56-FAM/-CCGCTACCAGTGATCACCAA |
| **20-comp** | TTGGTGATCACTGGTAGCGG |
| **25-FAM** | /56-FAM/-CCGCTACCAGTGATCACCAATGGAT |
| **25-comp** | ATCCATTGGTGATCACTGGTAGCGG |
| **30-FAM** | /56-FAM/-CCG CTA CCA GTG ATC ACC AAT GGA TTG CTA - |
| **30-comp** | TAGCAATCCATTGGTGATCACTGGTAGCGG |
| **49-FAM** | /56-FAM/ -TGG CGA CGG CAG CGA GGC TCT CTA CAG GAG CCT GTT AAG TGC TTG TAA C |
| **49-comp** | TTA CAA GCA CTT AAC AGG CTC CTG TAG AGA GCC TCG CTG CCG TCG CCA-3’ |
| **dT30-FAM** | /56-FAM/-(30)T |
| **dT49-FAM** | /56-FAM/-(49)T |
| **dT71-FAM** | /56-FAM/-(71)T |
| **JYM1413** | GGGCGAATTGGGCCCGACGTCGCATGCTCCTCTAGACTCGAGGAATTCGGTACCCCGGGTT  CGAAATCGATAAGCTTACAGTCTCCATTTAAAGGACAAG |

**Table D.** Substrates for strand exchange activity and FRET assays.

| **Sample** | **Sequence** |
| --- | --- |
| **35mer-Cy5** | AGGTCTTGTTCGCAGATGGCTTAGAGCTTATTTGC-/Cy5Sp/ |
| **35mer-IA-comp** | /IA/-GCAAATAAGCTCTAAGCCATCTGCGAACAAGACCT |
| **35mer-Cy3** | AGGTCTTGTTCGCAGATGGCTTAGAGCTTATTTGC-/Cy3Sp/ |
| **35mer-Cy5-comp** | /CY5/-GCAAATAAGCTCTAAGCCATCTGCGAACAAGACCT-3’ |
| **ss90-1 (for ds35)** | GCCTCTAGTCGAGGCATCAATACGAAACCTTATTCTTTCAGTCT  ACAAGCACTTAAGGTCTTGTTCGCAGATGGCTTAGAGCTTATTT  GC |
| **49mer-FAM** | /6FAM/-TGG CGA CGG CAG CGA GGC TCT CTA CAG GAG CCT GTT AAG TGC TTG TAA |
| **49mer-DAB-comp** | GTT ACA AGC ACT TAA CAG GCT CCT GTA GAG AGC CTC GCT GCC GTC GCC A-/DAB/ |
| **ss90-2 (for ds49)** | CACTTAAGGTCTTGTTCGCAGATGGCTTAGAGCTTATTTGCGTT ACAAGCACTTAACAGGCTCCTGTAGAGAGCCTCGCTGCCGTCGCCA |
| **RNA60** | TACGAAACCTTATTCTTTCAGTCTACAAGCACTTAAGGTCTTGTT  CGCAGATGGCTTAGAGCTTATTTGC |
| **Cy3-dT70-Cy5** | /Cy3Sp/-dT_70_-/Cy5Sp/ |
| **Cy3-ss40-Cy5** | /Cy5Sp/-ATAAGAGGTCATTTTTGCGGATGGCTTAGAGCTTAATTGC-/Cy5Sp/ |

**Table E.** siRNA resistance primers:

| JYM3892 | gatCTTATTGTTCTACCAGGAAAATC |
| --- | --- |
| JYM3893 | ttccTCTAAGTCCTCCATTTCTG |
| siRNA target sequences | |
| siCTL | UUCGAACGUGUCACGUCAA |
| siPALB2 | CUUAGAAGAGGACCUUAUU |
