# Supplementary material for: Novel RNA and DNA strand exchange activity of the PALB2 DNA binding domain and its critical role for DNA repair in cells
Source: eLife. 2019 Apr 29;8:e44063. doi: 10.7554/eLife.44063 (PMC6533086; doi:10.7554/eLife.44063)
Supplement: Transparent reporting form [file elife-44063-transrepform.pdf]

eLIFE
1st Floor
24 Hills Road
Cambridge CB2 1JP, UK
P 01223 855340
W elifesciences.org
T @elife

eLife's transparent reporting form

We encourage authors to provide detailed information *within their submission* to facilitate the interpretation and replication of experiments. Authors can upload supporting documentation to indicate the use of appropriate reporting guidelines for health-related research (see EQUATOR Network), life science research (see the BioSharing Information Resource), or the ARRIVE guidelines for reporting work involving animal research. Where applicable, authors should refer to any relevant reporting standards documents in this form.

If you have any questions, please consult our Journal Policies and/or contact us: editorial@elifesciences.org.

Sample-size estimation

- You should state whether an appropriate sample size was computed when the study was being designed
- You should state the statistical method of sample size computation and any required assumptions
- If no explicit power analysis was used, you should describe how you decided what sample (replicate) size (number) to use

Please outline where this information can be found within the submission (e.g., sections or figure legends), or explain why this information doesn't apply to your submission:

N/A

Replicates

- You should report how often each experiment was performed
- You should include a definition of biological versus technical replication
- The data obtained should be provided and sufficient information should be provided to indicate the number of independent biological and/or technical replicates
- If you encountered any outliers, you should describe how these were handled
- Criteria for exclusion/inclusion of data should be clearly stated
- High-throughput sequence data should be uploaded before submission, with a private link for reviewers provided (these are available from both GEO and ArrayExpress)

Please outline where this information can be found within the submission (e.g., sections or figure legends), or explain why this information doesn't apply to your submission:

Figs. 1,2 –Results are summary of 2 experiments with 3 replicates each.
Fig. 3A – representative images for Fig. 3B
Fig. 3B - RAD51 foci formation was scored with Volocity software v6.0.1 (Perkin-Elmer Improvision) using N=100 A-positive cells were used in dot plot representing SEM.
Fig. 3C - Data are represented as mean percentages of mClover-positive cells over the iRFP-positive population from five independent experiments (total n>100 iRFP-positive cells) and reported in a scatter dot plot representing SEM.
Figs. 4,5,7 – Plotted data were obtained from a single experiment with all proteins samples simultaneously measured in a plate reader with the identical substrate under identical conditions. Similar experiments with different set of proteins and mutants were performed at least 10 times for each sample using at least 3 different protein purifications.

Statistical reporting

- Statistical analysis methods should be described and justified
- Raw data should be presented in figures whenever informative to do so (typically when N per group is less than 10)
- For each experiment, you should identify the statistical tests used, exact values of N, definitions of center, methods of multiple test correction, and dispersion and precision measures (e.g., mean, median, SD, SEM, confidence intervals; and, for the major substantive results, a measure of effect size (e.g., Pearson's r, Cohen's d)
- Report exact p-values wherever possible alongside the summary statistics and 95% confidence intervals. These should be reported for all key questions and not only when the p-value is less than 0.05.

Please outline where this information can be found within the submission (e.g., sections or figure legends), or explain why this information doesn't apply to your submission:

Figs. 1,2 – Data were fit using a standard four-parameter logistic fit (Prism).
Fig. 3B - N=100 A-positive cells were used in dot plot representing SEM. An Anova test (Kruskul-Wallis test for multiple comparison) was performed followed by a non-parametric Mann-Whitney test.
Fig. 3C - a classical one-way Anova test was performed.

(For large datasets, or papers with a very large number of statistical tests, you may upload a single table file with tests, Ns, etc., with reference to sections in the manuscript.)

Group allocation

- Indicate how samples were allocated into experimental groups (in the case of clinical studies, please specify allocation to treatment method); if randomization was used, please also state if restricted randomization was applied
- Indicate if masking was used during group allocation, data collection and/or data analysis

Please outline where this information can be found within the submission (e.g., sections or figure legends), or explain why this information doesn't apply to your submission:

N/A

Additional data files ("source data")

- We encourage you to upload relevant additional data files, such as numerical data that are represented as a graph in a figure, or as a summary table
- Where provided, these should be in the most useful format, and they can be uploaded as "Source data" files linked to a main figure or table
- Include model definition files including the full list of parameters used
- Include code used for data analysis (e.g., R, MatLab)
- Avoid stating that data files are "available upon request"

Please indicate the figures or tables for which source data files have been provided:

